# Relationship between 8/9-yr-old school children BMI, parents' BMI and educational level: a cross sectional survey

**DOI:** 10.1186/1475-2891-10-76

**Published:** 2011-07-19

**Authors:** Giacomo Lazzeri, Andrea Pammolli, Valentina Pilato, Mariano V Giacchi

**Affiliations:** 1 CREPS-Research Center for Health Education and Promotion, University of Siena, Via A. Moro 2 Siena 53100, Italy

**Keywords:** childhood, obesity, underweight, parents' nutritional status, educational level

## Abstract

**Background:**

Parents are responsible not only for the genetic structure of their children, but also for passing onto them their behaviours and attitudes toward life. The aim of this study was to analyse the connection between school-age children's obesity and that of their parents as well as between child obesity and parents' educational level, as a proxy indicator of the socio-economic status (SES) of families in Tuscany.

**Methods:**

The children sample was selected from **"**OKkio alla Salute 2010" (a cross sectional survey carried out by the Italian Institute of Health) and consisted of 1,751 (922 males and 855 females) 8-9 year-old school children. Weight and height were measured by ad hoc trained personnel, and Body Mass Index (BMI) categories were calculated using Cole *et al*.'s cut-off. Parents' weight, height and educational level were collected by a self-administered questionnaire. The educational levels were classified as high, medium and low.

**Results:**

The prevalence of obese children increased along the parents' BMI category: from 1.4% for underweight mothers to 30.3% for obese mothers and from 4% for under-normal-weight fathers to 23.9% for obese fathers (p < 0.001). An inverse relationship was observed between the parents' educational level and child obesity, the lowest educational level corresponding to the highest prevalence of obese children: 9.3% for mothers with a low educational level compared to 5.8% for mothers with a high educational level (p = 0.15); similarly, the corresponding prevalence for fathers was 9.5% compared to 4.5% (p = 0.03).

**Conclusion:**

Parents' obesity and the cultural resources of the family, particularly the father's, seem to influence the prevalence of overweight and obesity in Tuscan children.

## Background

The prevalence of childhood obesity is increasing rapidly in industrialized nations. Childhood obesity can create many complications at the cardiovascular, endocrine, pulmonary, musculoskeletal and gastrointestinal levels, as well as possible psycho-social consequences (poor self esteem, depression, eating disorders) [1]. For this reason, recent studies have focused on the possible causes and risk factors associated with obesity in paediatric ages. Obesity is known to be a multi-factorial disorder originating from the interaction of genetic and environmental factors; the accumulation of body fat is a very complex phenomenon regulated by a series of physiological mechanisms, some of which are still unknown. Therefore, the genetic factor in families seems to play an important role in the risk of obesity for a child, although it only partly explains the phenomenon [2-5]. Numerous studies have shown that the risk of becoming obese is higher in children of obese parents [6-9]. Parents are responsible not only for the genetic structure of their children, but also for passing onto them their behaviours and attitudes toward life. The lifestyle of the family plays an important role in the nutritional and behavioural choices of the child and this can be the result of both social and economic factors, such as the place of residence; the parents' cultural level; the economic assets of the family; and their income. It is well known that there is an inverse relationship between socio-economic conditions and the health status of the population in developed countries. In Italy, it has been shown that people from a low socio-economic level are subject to a higher risk of morbidity and mortality [10,11]. A study by Mackenbach JP *et al*. showed that, during the 1990s, in the six countries considered (Finland, the Netherlands, Denmark, the United Kingdom, Sweden and Italy), the differences in mortality rates among population groups in high and low socio-economic conditions were growing, compared with an overall reduction in mortality rates [12]. Studies have shown an inverse relationship between children Body Mass Index (BMI) and family educational level; in this context, the parents' educational level is one of the most commonly used indicator [13-15].

In the context of the program "Gaining Health in Europe" launched by WHO-Europe in 2006, the first population based nutritional surveillance system named "OKkio alla Salute" was launched in Italy.

The aim of the present study is to focus on the risk factors for obesity in paediatric age and, particularly, on the possible association between parental and child obesity and the influence that parents' educational level could exert on children's weight status.

## Methods

The protocol for the "Okkio alla Salute" survey developed by the National Institute of Health and reviewed in collaboration with regional coordinators and members of the technical committee is described elsewhere [16].

In this study we will report data regarding the 2010 Tuscany Regional project. In our region, to guarantee the maximum level of territorial coverage, all 12 Local Health Units were invited, and all agreed to join, and collaborate in, the project. Once enrolled, all 12 Local Health Units met for an explanation of the protocol and to arrange the operational formalities of the activities in Tuscany.

Overall, opt-out consent was signed by 95.5% (1983 children) of the parents of the children enrolled in the 99 classes selected; 91.3% (1811 children) of all invited children were present on the day of the study. After data cleaning, 60 more children were discarded (34 out of age range or with missing age); the remaining 88.3% (1,751 children: 922 males and 855 females) out of all enrolled children were analysed.

Students were selected by a stratified one-stage sample with classes as clusters of students and primary stratification by relative health district and municipality size. The number of children to be enrolled was estimated on the basis of an expected prevalence of overweight and obesity of 30%, a precision of 3% using a 95% confidence intervals, and a design effect of 2 [16].

Specifically trained personnel using appropriate and standardised instruments measured the children's height and weight. To measure the anthropometric values, we followed the recommendations of the World Health Organization [17].

We used electronic scales with a liquid crystal display that measured every 100 grams of weight. Height was measured with a portable stadiometer, with a precision of 0.1 cm; exact decimal age was calculated from the date of birth and day of measurement; BMI was then calculated from weight and height, using the following formula: weight (kg)/height (m²). BMI classes of the children were set using the Cole *et al*. Method [18,19]; this allowed us to have specific cut-off points for males and females at every age as recommended by the International Obesity Task Force (IOTF). We thus obtained six classes of BMI: thinness grades 3, 2, 1, normal weight, overweight and obesity. According to the Cole's definition, the term "underweight" in children includes thinness grades 3 and 2 (underweight group) while thinness grade 1 and normal weight go into another class (normal group) [19] as shows in table 1.

**Table 1:** Nutritional status of 8-9 year old school-children

		**No**.	%	IC_95%_
***Underweight***	**Thinness 3** **Thinness 2**	6 5	0.3 0.3	0.06-0.63 0.02-0.54
***Normal***	**Thinness 1** **Normal**	91 1113	5.4 64.3	4.24-6.53 61.96-66.63
***Overweight***	**Pre-obese**	387	21.8	19.73-23.92
***Obese***	**Obese**	149	7.9	6.54-9.19

	**Total**	1751	100	

The weight, height and educational level of both parents were recorded in a self-administered questionnaire. Weight (kg) and height (m) were used for the calculation of the BMI (kg/m²); the parents' BMI classes (underweight, normal weight, overweight or obese) were established using international cut-off points for adults [17]. For 132 (7.4%) mothers and 202 (11.4%) fathers the BMI calculation was not possible due to lack of the necessary data.

Three levels of education were then established: high (university degree), medium (secondary school diploma) and low (middle school, elementary school or none).

Data were analyzed using SPSS (ver.16) and EpiInfo (ver.3.5.1.). Descriptive statistics (e.g., mean, proportion, standard deviation) were used to describe the characteristics of the sample. The χ^2^-test and χ^2^-test for trend were used to explore the relationship between: a) children's BMI classes and parents' BMI classes, b) children's BMI classes and parents' educational level.

The Kendall's τ_b _coefficient was used to calculate the measure of association between children's BMI classes and parents' BMI classes and children's BMI classes and their parents' educational level.

The association between children's obesity and their parents' educational level was estimated comparing the Prevalence Ratios (PR), of children whose mothers and fathers had a low educational level with that of children born from mothers and fathers with a high educational level.

All analyses were carried out using the C-Sample routines for complex survey design.

## Results

**Figure 1:**
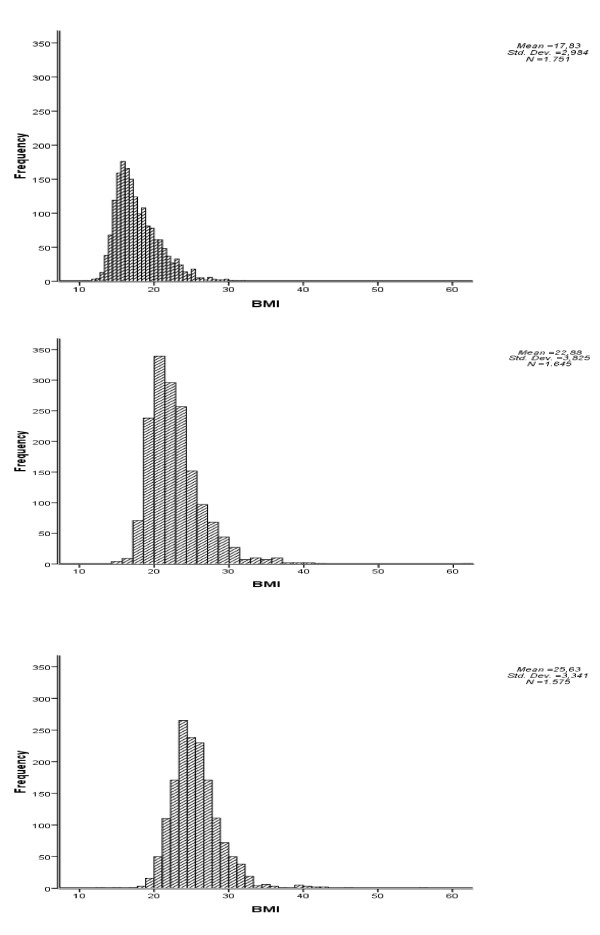
**Frequency of children, mothers and fathers' BMI values**.

Thanks to the participation of all the Local Health Units, it was possible to produce a representative sample of the population of 8/9-year-old Tuscan children. The frequency of BMI values, BMI means ± standard deviation of children, mothers and fathers is described in Figure 1. The prevalence of overweight and obesity in children is 21.8% and 7.9% respectively (table 1).

Table 2 shows the distribution of the parents' BMI classes. It is evident that there are more overweight (44.0%) and obese (8.3%) fathers compared to mothers (17.3% and 4.1%). There is also a clear prevalence of underweight mothers compared to fathers (5.0% vs. 0.4%).

**Table 2:** Distribution of parents' BMI class

	Mother			Father		
BMI Class	**No**.	%	95% CI	**No**.	%	95% CI
Underweight	78	5.05%	3.8-6.2	5	0.36%	0.05-0.68
Normalweight	1212	**73.59%**	71.1-76.1	743	**47.33%**	44.3-50.3
Overweight	285	**17.3%**	15.2-19.4	692	**43.99%**	41.3-46.6
Obese	70	**4.06%**	3.0-5.1	135	**8.32%**	6.8-9.8

Total	1645	**100%**		1575	**100%**	

Missing	132/1777			202/1777		

If we relate the BMI class of the child with that of the mother and father (table 3, 4) we observe, in both cases, that the prevalence of obese children increases with increasing parents' BMI classes (p = 0.01). Furthermore, the prevalence of obese children of obese mothers (30.3%) is higher than that observed in children of obese fathers (23.9%), but the difference is not statistically significant (p = 0.3).

**Table 3:** Children's BMI class compared to mother' BMI class

	CHILD				
	
	Underweight^a^ (%)	Normalweight^b ^(%)	Overweight (%)	Obese (%)	Total No. (%)
**MOTHER**					
Under-normal weight	0.8	74.2	20.0	5.0	1273 (100)
Overweight	0.4	56.1	29.9	13.6	279 (100)
Obese	----	48.2	21.4	30.3	68 (100)

Total No.	11	1119	358	132	1620

Chi square for trend	p = 0.24	p < 0.001	p = 0.046	p < 0.001	
Kendall's τ**_b_**					p < 0.001

^a ^The underweight class include thinness 2 and 3.

^b ^The Normal class include normal and thinness 1.

**Table 4:** Children's BMI class compared to father's BMI class

	CHILD				
	
	Underweight^a^ (%)	Normalweight^b ^(%)	Overweight (%)	Obese (%)	Total No. (%)
**FATHER**					
Under-normal weight ^c^	0.9	76.3	18.8	4.0	722 (100)
Overweight	0.3	67.1	24.2	8.3	680 (100)
Obese	0.9	51.1	24.1	23.9	133 (100)

Total No.	10	1062	338	125	1535

Chi square for trend	p = 0.28	p < 0.001	p = 0.01	p < 0.001	
Kendall's τ_b_					p < 0.001

^a ^The underweight class include thinness 2 and 3.

^b ^The Normal class include normal and thinness 1.

^c ^The underweight class was linked to the normal weight class because numerically not significant

The weighted prevalence of obese children of mothers with an excess of weight (overweight plus obese) is 16.7%, derived from raw data presented in tables 3, while the corresponding value of children from fathers with an excess of weight (table 4) is 10.8%; the difference results statistically significant (p = 0.006).

**Figure 2:**
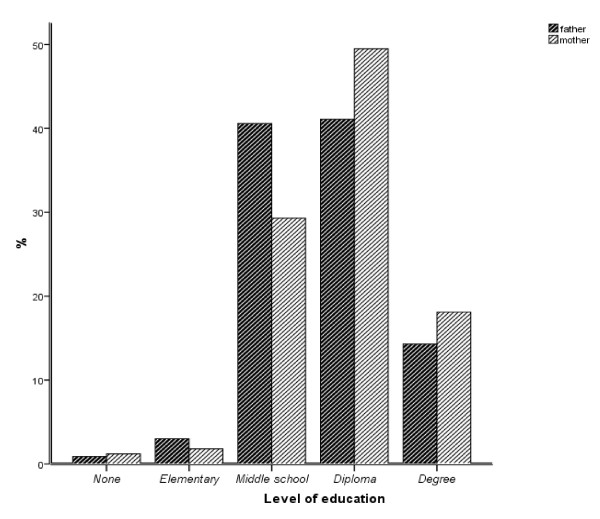
**Parents' educational level**.

Most of the parents have a middle or secondary school diploma; the most frequent level of education of mothers and fathers is the secondary school diploma. The percentage of mothers with a High School diploma (49.5%) and with a University degree (18.6%) is higher than that of fathers (41.1% and 14.4% respectively) (Figure 2).

The analysis of the relationship between the parents' educational level and the BMI class of their children (table 5) shows that the lowest percentage of obesity (2.8%) and the highest percentage of normal-weight (82.3%) correspond to the parents' level of education "Mother MEDIUM-father HIGH"; vice-versa, the highest percentage of obesity (10.6%) and one of the lowest percentage of normal-weight (66.3%) correspond to the parents' level of education "Mother LOW-father MEDIUM".

**Table 5:** Distribution of children's BMI by parents' educational level

		Children's BMI class			
	
Parents educational level	**No**.	Underweight (%)	Normalweight (%)	Overweight (%)	Obese (%)
Mother and father HIGH	126	0.2	72.8	21.8	5.2
Mother HIGH-father MEDIUM	125	----	66.6	27.5	5.9
Mother HIGH-father LOW	40	2.81	64.8	23.9	8.4
Mother MEDIUM-father HIGH	79	----	82.3	14.8	2.8
Mother and father MEDIUM	402	0.07	68.8	24.6	6.5
Mother MEDIUM-father LOW	310	1.1	68.9	20.5	9.5
Mother LOW-father LOW	347	0.99	69.7	19.6	9.7
Mother LOW-father MEDIUM	118	0.96	66.3	22.2	10.6

Total	1563	0.63	69.5	22.0	7.8

p					<0.001^a^

Mother low - father high (n = 16) are not included due to their low number.

^a ^Chi square (not considered underweight class)

This data is also confirmed by the separate analysis for the fathers' and the mothers' level of education (table 6, 7). It is thus evident that the prevalence of obese children is inversely related to the educational level of fathers. In fact, the percentage varies from the highest value of 9.5% of obese children from fathers with a low educational level to the lowest value of 4.5% of obese children from fathers with a high educational level (p = 0.03); for mothers the inverse relationship is present (from 9.3% to 5.8% respectively) but the difference is not statistically significant (table 8).

**Table 6:** Relationship between children's BMI class and father' educational level

	CHILD				
	
Educational level	Underweight (%)	Normalweight (%)	Overweight (%)	Obese (%)	Total No. (%)
**FATHER**					
*Low*	1.1	69.4	20.0	9.5	704 (100)
*Medium *	0.2	68.0	24.7	7.1	653 (100)
*High*	0.1	75.5	19.9	4.5	223 (100)

Total No.	10	1087	352	131	1580

Chi square for trend	p = 0.03	p = 0.07	p = 0.12	p = 0.03	
Kendall's τ**_b_**					p = 0.012

**Table 7:** Relationship between children's BMI class and mother' educational level

	CHILD				
	
Educational level	Underweight (%)	Normalweight (%)	Overweight (%)	Obese (%)	Total No. (%)
**MOTHER**					
*Low*	0.8	69.1	20.8	9.3	534 (100)
*Medium*	0.6	70.0	21.9	7.5	837 (100)
*High*	0.5	69.8	23.9	5.8	298 (100)

Total No.	11	1147	371	140	1669 (100)

Chi square for trend	p = 0.76	p = 0.93	p = 0.59	p = 0.15	
Kendall's τ**_b_**					p = 0.19

When calculating the PR's of obese children by the parents' educational level, from the data reported in tables 7, we find that the PR of children whose mothers have a low educational level, compared to children born from mothers with a high educational level, is 1.6 (IC_95% _0.93-2.69). The PR of children whose fathers have a low educational level compared to children born from fathers with a high educational level is 2.1 (IC_95% _1.11-4.11).

## Conclusion

Parents' obesity and the cultural resources of the family, particularly the father's, seem to influence the prevalence of overweight and obesity in Tuscan children.

Many studies in the field of obesity in school-aged children have emphasized the role of the family. Parent's genetic and behaviour factors, which are, consciously or unconsciously, transmitted to their children, are essential to explaining the nutritional status of these children [20,21].

In the present study we should emphasise that weight and height used to calculate the BMI, are directly measured in children while parents' weight and height are self-reported.

The parents' obesity is certainly one of the most important factors in favouring an increase of the children's weight and obesity [8,22]. In fact, the relationship between the children and the parents' BMI is significant. The parents are also responsible for the quality and the availability of food within the home and, for this reason, if their food habits are incorrect those of their children will follow [23]. A study by Wardle J. *et al*. [24] compared the food and activity preferences and lifestyles of children, independently of their BMI classes, from obese and lean families. The results showed that: "Children from the obese/overweight families had a higher preference for fatty foods in a taste test, a lower liking for vegetables, and a more 'overeating-type' eating style. They also had a stronger preference for sedentary activities, and spent more time in sedentary pastimes. There were no differences in speed of eating or reported frequency of intake of high-fat foods".

The analysis of educational levels shows that there is an inverse relationship between the parents' educational level (considered both together and separately) with overweight and obesity in 8- to 9-year-old children in Tuscany, similar to what has been found in other studies in Northern Italian pre-pubertal age children [15]. In particular, regarding the educational level, the influence of the father is stronger than that of the mother. In fact, analysing the parents together, among the various combinations that support this assumption, one is particularly evident; in the case of the mother at a medium and the father at a high educational level, only 2.8% of the children were obese, whilst in the opposite case, with the father at a medium and the mother at a low level, the percentage rose to 10.6%. If we evaluate the parents' educational levels separately, we find that fathers have a stronger influence on the prevalence of obesity in their children (PR of obesity in children born from fathers with low educational level is 2.1; category of reference: high educational level). These considerations are coherent with previous studies on the association between diet and physical activity with parent's social economic level.

It has been demonstrated that the diet of groups in a low socio-economic level is characterized by cheap foods, poor in nutrients and reach in high energy density (fats, sugars, full cream milk, preserves, potatoes and cereals, meat products) [25,26]; on the other hand, it has been observed that the father's educational level is directly associated with sports activities in schoolchildren [27].

In conclusion, we can state that, beyond the genetic predisposition in which the role of parents is clearly identifiable, it is possible, at least in Tuscany Region, that the family's low educational level, both in terms of economic and cultural resources, prevalently of the father, favours the increase of weight in school-age children.

Furthermore, it can be hypothesized, certainly after additional analyses, that parents' obesity and family SES can be utilized as potential predictors for a child becoming obese and remaining so over time.

Following the previous considerations We would like to point out that the target population to be firstly involved in health promotion and education programs should be the families with a low educational level.

## Competing interests

The authors declare that they have no competing interests.

## Authors' contributions

GL wrote the manuscript and organized the data collection; AP performed statistical analyses; VP collaborated to organize the data collection and input; MVG collaborated to the outline of the study, and to the final review of the manuscript. All authors read and approved the final manuscript.
